# Überzeugungen im Zeitenwandel

**DOI:** 10.1007/s00115-024-01659-9

**Published:** 2024-04-16

**Authors:** Hanfried Helmchen

**Affiliations:** Klinik für Psychiatrie und Psychotherapie, CBF, Charité – Universitätsmedizin Berlin, Hindenburgdamm 30, 12200 Berlin, Deutschland

Der tiefgreifende Wandel der gesellschaftlichen Atmosphäre lässt fragen, wie sich individuelle Überzeugungen gegenüber den gesellschaftlich herrschenden Ideen („Zeitgeist“) verhalten und inwieweit sie einander bedingen. So können individuelle Überzeugungen zu gesellschaftlich herrschenden Ideen werden, wenn sie von charismatischen Individuen vorgetragen werden und Menschen enthusiasmieren. Ebenso geht es umgekehrt um die Frage, wie weit individuelle Überzeugungen sich dem Wandel des Zeitgeistes anpassen oder stabil bleiben – und dadurch auch widerständig werden können. Anpassung kann Zeichen von Lernfähigkeit sein, sich durch Argumente überzeugen zu lassen. Anpassung kann aber auch auf Opportunismus hinweisen, wenn man sich um des eigenen Vorteils willen (einschließlich des Wohlfühlens in einer irrenden Gruppe) an unerwünschte gesellschaftliche Veränderungen anpasst. Nicht selten sind beide Determinanten von Anpassung gemischt. Ebenso kann es positiv als charakterfest konnotiert werden, an der eigenen Überzeugung trotz eines dagegenstehenden Zeitgeistes festzuhalten; das gleiche Verhalten kann aber auch als Lernunfähigkeit oder Rigidität eines „Betonkopfes“ denunziert werden.

Gelegentlich bleibt offen, ob einer Anpassung an den Zeitgeist ein Überzeugungswandel oder nur ein taktisches Überlebensmotiv zugrunde liegt. Zwei Beispiele:

## Karl Birnbaum (1878–1950).

Karl Birnbaum [1] arbeitete in der psychiatrischen Heil- und Pflegeanstalt Berlin-Herzberge, habilitierte sich an der Berliner Universität, wurde 1930 Direktor der Heil- und Pflegeanstalt Berlin-Buch, 1933 von den Nationalsozialisten seines Amtes „aus rassischen Gründen“ enthoben und emigrierte 1939 in die USA [2]. Gleichwohl konnte er – und auch das gilt tragischerweise für viele andere ebenso – dem Einfluss des Zeitgeistes auf sein Denken nicht ganz entgehen, wenn er in seinem 1935 bei Springer erschienenen populärwissenschaftlichen Buch „Die Welt des Geisteskranken“ [3] schreibt, dass „… die aufs engste kulturverbundene besondere Fürsorge für alle Art Hilflose und Unzulängliche, insbesondere auch für die psychisch *Defekten* …“ der Auslese entgegenwirkt, „mit der die Natur selbst alles *Lebensunwerte*, biologisch *Minderwertige* und Kranke aus dem Lebensprozess auszuschalten pflegt, und“ damit eine *Gegenauslese* fördert, „bei der Familien mit seelisch unzulänglichen Mitgliedern den gleichen, wenn nicht etwa gar einen größeren Anteil an der Fortpflanzung behalten können wie die Gesunden und *Vollwertigen*.“ (Birnbaum 1935, kursiv hervorgehoben durch H.H.).

Hat Birnbaum sich hier nur der Sprache seiner nationalsozialistischen Umgebung bedient, um sein Buch publizieren zu können oder weitergehenden Repressionen zu entgehen? Oder veranschaulicht das tragische Schicksal Birnbaums, wie stark der Zeitgeist die Sprache eines Menschen anscheinend unreflektiert durchdringt, der die Auswüchse eben dieses Zeitgeistes erleiden muss?^1^

## Franz Kallmann (1897-1965).

Franz Kallmann [1, 5] erwarb seine Facharztqualifikation bei Karl Bonhoeffer und wurde 1928 Leiter der Abteilung für Neuropathologie ebenfalls in der Anstalt Herzberge. Seine psychiatrische Gutachtertätigkeit regte ihn an, Daten von Familien mit schizophren erkrankten Mitgliedern zu sammeln, um deren erbbedingten Hintergrund mit der von Ernst Rüdin an der demographisch-genetischen Abteilung der Forschungsanstalt für Psychiatrie in München entwickelten genetischen Methodik weitergehend zu prüfen. In diesem Münchener Arbeitskreis verbrachte er von 1931 bis 1936 in jedem Jahr einige Wochen, um mit den Kollegen zusammen zu arbeiten und sein methodisches Wissen zu aktualisieren. Seine vorläufigen Untersuchungsergebnisse insbesondere zur Fertilität von Menschen mit Schizophrenie und ihren Familienmitgliedern konnte er noch auf dem International Congress of Population Science in Berlin 1935 vortragen, durfte dies als (getaufter) Jude aber nicht mehr am Ende desselben Jahres auf dem Kongress der Gesellschaft Deutscher Neurologen und Psychiater. Stattdessen wurden seine Befunde von seinem Münchener Kollegen Bruno Schulz vorgetragen, aber im Kongressband unter Kallmanns Namen publiziert – auch, wie Volker Roelcke ausführt, ein Hinweis darauf, dass seine eugenischen Schlussfolgerungen der in Deutschland herrschenden rassenhygienischen Ideologie entsprachen. 1936 flüchtete Kallmann nach New York, publizierte ab 1938 die Ergebnisse seiner Familien- und Zwillingsstudien zur Schizophrenie, wurde 1954 zum Professor für Psychiatrie an der Columbia University ernannt, gründete die erste Abteilung für psychiatrische Genetik in den US und wurde ein anerkannter Pionier der psychiatrischen Genetik. Seine Befunde fanden schlussendlich große Resonanz und blieben bis in die 1980er-Jahre der wichtigste Beleg für eine erbliche Komponente der Schizophrenie. Aber er vertrat weiter seine eugenische Überzeugung, Menschen mit Schizophrenie und deren Familien einer Fortpflanzungskontrolle zu unterwerfen, um einer Verschlechterung des Gen-Pools entgegenzuwirken – zum Wohle und zur Sicherheit der Allgemeinheit. Allerdings argumentierte er dabei nicht mehr für gesetzliche Regelungen zur Zwangssterilisation und verzichtete auf Begriffe wie Rasse und Volkskörper, sondern sprach sich nun vielmehr für genetische Beratung und freiwillige Sterilisation aus und betonte, dass die Manifestation der genetischen Komponente der Schizophrenie von sozialen und psychischen Faktoren beeinflusst werde. Ob darin mehr als nur eine rhetorische Anpassung an Werte der ihn umgebenden amerikanischen Gesellschaft zum Ausdruck kommt, bleibt zweifelhaft. Denn einerseits lebte er in den ersten 15 Jahren in den USA in ungesicherten Verhältnissen und musste sich ständig um Drittmittel für seine Forschungsarbeit bemühen, hielt aber andererseits unverändert an der eugenischen Zielsetzung seiner Konzeption fest, wenn er sich auch von der Zwangssterilisation distanzierte und stattdessen auf „die umfassende genetische Beratung der Betroffenen setzte“ und sich damit „dem neuen eugenischen Diskurs anpasste“.^2^

Kallmanns Entwicklung in den liberal-demokratischen USA zeigt, dass und wie weit sich ein Individuum dem herrschenden Geist seiner soziokulturellen Umgebung anpasst, wobei es letztlich nachrangig ist, ob die Motive opportunistisch sind oder in veränderten oder neuen Überzeugungen liegen.

Solche Anpassungen sind nicht nur individuell, sondern auch für ganze Gesellschaften zu beobachten, wenn sich der epochale Hintergrund ändert. So lautete vor 200 Jahren der erste Satz eines verbreiteten und einflussreichen Psychiatriebuches: „ein Irrer ist wie ein unmündiges Kind“ [8]. – Heute hingegen fordert der Respekt vor den Menschenrechten, das Selbstbestimmungsrecht auch von Menschen mit psychischen Krankheiten anzuerkennen. Aber auch nach einem kürzeren Zeitraum ist solch ein Einstellungswandel deutlich. So waren Mitte des vorigen Jahrhunderts junge Ärzte empört, wenn sie zufällig hörten, dass Juristen ihre ärztliche Intervention mit Strafe bedrohten, wenn sie nicht die Zustimmung des Patienten eingeholt hätten. Empört waren sie, weil sie überzeugt waren, dass ihre wissensbasierte fachliche Kompetenz besser als die des Patienten war und dass ihr darauf gegründetes Handeln dem Besten ihres Patienten diente. Gelegentlich wurde dies Patienten deutlich zu machen gesucht, indem man ihnen sagte, dass sie zwar die Experten für das Kranksein sind, also für das Leiden an der Krankheit, der Arzt hingegen der Experte für die Krankheit ist, die er behandeln soll. Heute jedoch informieren Ärzte den Patienten vor jeder Intervention über deren Nutzen und Risiken und unterstützen seine Entscheidung – und sind zunehmend davon überzeugt, dass dieser Wandel von paternalistischer zu partnerschaftlicher Einstellung richtig ist.

Das führt zur Frage nach dem Umfeld, in dem sich unsere heutigen Überzeugungen entwickelt haben? Gemeint ist der *gesellschaftliche* Kontext

- vom nationalsozialistischen Terrorstaat über
- das Schweigen der aufbauorientierten Nachkriegsgeneration
- zur änderungswillig-erregten Stimmung der 1968er-Jugendrebellion
- und weiter über die zeithistorisch unterlegte Entwicklung unserer Erinnerungskultur
- bis zur heutigen liberal-demokratischen Gesellschaftsverfassung auf der Grundlage eines Grundgesetzes, das unmittelbar nach dem Krieg aus der Diktaturerfahrung geboren worden war.

*Psychiatriespezifischer* wurde der Kontext bestimmt

- *inhaltlich* durch die Aufbruchstimmung der 1950er-Jahre, mit neuen Arzneimitteln wirksam behandeln zu können und mit der ambulanten Langzeitmedikation auch den sozialen Kontext der Patienten in den Blick zu bekommen [9],
- *regulativ* durch die Entwicklung klinischer Prüfmethodik zur Messung ihrer klinischen Wirksamkeit bis hin zu ihrer juristischen Fixierung im Arzneimittelgesetz (AMG) in den 1960er-Jahren,
- *ethisch *durch das Auftauchen moralischer Fragen wie dem ethischen Paradoxon [10, 11] und die Sensibilisierung für ethische Fragen klinischer Forschung durch die Einrichtung von Ethikkommissionen ab den 1970er-Jahren,
- *zeithistorisch* durch die Vertiefung ethischer Fragen ab den 1980er-Jahren unter dem Eindruck der zunehmenden öffentlichen Wahrnehmung zeithistorischer Aufdeckung der nationalsozialistischen Verbrechen wie der Zwangssterilisation und der Tötung psychisch kranker Menschen [12].

Vermutlich viele Ärzte hatten zwar Mitscherlichs und Mielkes Bericht (1960; [13]) über die bereits im Nürnberger Ärzteprozess 1948 aufgedeckten Verbrechen gelesen, sie aber nur einzelnen Ärzten oder einer nur sehr kleinen Minderheit von Ärzten zugeordnet. Etliche waren gewiss auch entsetzt, konnten jedoch keinen Bezug zu ihrer ärztlichen Tätigkeit sehen. Erst ab den 1990er-Jahren wurde die Relevanz dieser damals verurteilten Verbrechen für den heutigen Umgang mit psychisch kranken Menschen deutlich [14],

- so etwa die damals euphemistisch *„Euthanasie“* bzw. „Gnadentod“ genannte und gleichwohl geheim gehaltene Ermordung psychisch Kranker für den heutigen Umgang mit dem assistierten Suizid
- oder die *Zwangssterilisation* chronisch psychisch Kranker aus eugenischen oder ökonomischen Gründen, die heute bei Priorisierungen aus wirtschaftlichen Gründen nicht ausgeschlossen sind,
- oder auch die *potenziell tödlichen Experimente* mit Menschen ohne deren Einwilligung für die durch den Nürnberger Kodex angestoßene Entwicklung der heute gültigen differenzierten Regeln zur Forschung mit nicht einwilligungsfähigen Kranken.

Dabei wurde auch die Frage virulent: wie stabil unsere heutige Überzeugung ist, wenn sich der Zeitgeist weiterdreht, wenn etwa eine konfuzianische oder islamische oder auch uns noch unbekannte Gesellschaftsüberzeugung die im abendländischen Europa herrschende jüdisch-christliche Idee möglicherweise ersetzt haben könnte?

Wer das für abwegig hält, sollte daran denken, dass die gegen die christliche „Humanitätsduselei“ gerichtete rassenhygienische Ideologie der Nationalsozialisten ein Rückfall in vorchristlich-archaische Gesellschaftsverhältnisse war, ein Rückfall, den Hitler aktiv betrieb:

„Unsere Revolution ist nicht bloß eine politische und soziale, wir stehen vor einer ungeheuren Umwälzung der Moralbegriffe und der geistigen Orientierung des Menschen. Wir beenden einen Irrweg der Menschheit. Die Tafeln vom Berge Sinai haben ihre Gültigkeit verloren. Das Gewissen ist eine jüdische Erfindung“ [15, S. 210].

Mit den Tafeln vom Berge Sinai bezeichnete Hitler den Dekalog. Wenn diese Quelle des Zitats von Historikern auch für fragwürdig gehalten wird, so gibt es doch Hitlers ideologische Überzeugung wieder, die von Nationalsozialisten wie etwa Hans Frank, dem nationalsozialistischen Generalgouverneur des unter Reichsverwaltung gestellten Teils Ostpolens und Galiziens („Generalgouvernement“), übernommen und in die ungeheuerliche Wirklichkeit der Ermordung von 3,5 Mio. Juden umgesetzt wurde. Der Völkerrechtler Philippe Sands zitiert in seinem Buch „Rückkehr nach Lemberg“ (2016/18; [16]) Hans Frank:

„Der Nationalsozialismus hat das falsche Prinzip des Humanismus abgeschafft“ [16, S. 295].

Das Zitat stammt aus einer Rede Franks auf dem 11. Internationalen Kongress für Strafrecht und Gefängniswesen 1935 in Berlin. Geoffrey Bing, ein englischer Anwalt war als Teilnehmer entsetzt „über den Anblick von ausländischen Beamten, Kriminologen und Reformern, die Franks >monströse Vorschläge< bejubelten“ [16, S. 296]. 1941 äußerte Frank in Bezug auf die Wannsee-Konferenz zur „Endlösung der Judenfrage“:

„Wir müssen die Juden vernichten, wo immer wir sie treffen und wo es irgend möglich ist“ [16, S. 301].

Dieses Zitat stammt aus dem Diensttagebuch, in dessen 38 Bänden Frank seine Aktivitäten als Generalgouverneur detailliert aufzeichnen ließ; Sands fragte sich, ob Franks Sekretäre „sich jemals Gedanken darüber gemacht hatten, ob es klug sei, solche Äußerungen aufzuschreiben.“ Dies ist allerdings zu bezweifeln, da diese Vernichtungsideologie offenbar weite Kreise zog, wie die folgende Ausführung des „Reichsgesundheitsführers“ Leonardo Conti zeigt. Conti antwortete dem Göttinger Professor Gottfried Ewald, jenem Psychiatrieprofessor, der sich als einziger deutlich gegen die Tötung psychisch Kranker wandte. Conti schrieb:

„… ich bin fest überzeugt, daß die Anschauungen des ganzen deutschen Volkes in diesen Dingen in einer Wandlung begriffen sind, und kann mir sehr wohl vorstellen, daß Dinge, die in einer Zeitspanne als verwerflich gelten, in der nächsten als das einzig Richtige erklärt werden. Das haben wir im Laufe der Geschichte ja unzählige Male erlebt. Als letztes Beispiel kann ich ganz ruhig auf das Sterilisierungsgesetz verweisen; hier ist der Prozeß der Umformung des Denkens heute doch schon recht weit vorgeschritten“ [17, S. 56].^3^

Contis Antwort belegt, wie überzeugte Nationalsozialisten an diesem grundlegenden Wandel moralischer Normen arbeiteten. Die Zitate verdeutlichen Hitlers biologistisch-totalitäre Ideologie als Ausdruck eines Zeitgeistes, den der Soziologe Lepenies als gegenaufklärerisch charakterisierte [20, S. 69f.]. Eingebettet in jenen Zeitgeist verspottete Hitler den jüdisch-christlichen Humanismus als „Gefühlsduselei“ und setzte dessen Beseitigung mit emotionalisierten Massen um. Der rationale Gehalt dieses Humanismus wurde in der Formulierung der Straftatbestände „Verbrechen gegen die Menschlichkeit“ und „Genozid“ im Nürnberger Kriegsverbrecherprozess festgeschrieben [16] und wird in den Menschenrechten zunehmend konkretisiert.

## Fazit für die Praxis

Indem wir diesen Wandel des Zeitgeistes beobachten und erkennen, wie sehr wir „als Kinder unserer Zeit“ in ihn eingebettet sind, ist zu fragen, anhand welcher Kriterien wir erkennen können, dass der Zeitgeist uns in gefährliche Regionen zu verführen droht. Dabei erscheinen Ideologien oder Dogmen umso verführerischer, je mehr sie Elemente der Wirklichkeit enthalten. Dies sollten wir nicht erst erkennen, wenn uns die Menschenfeindlichkeit des Zeitgeistes berührt. Immerhin benennen die heute zahlreich entwickelten Ethikkodizes Kriterien, die zur Reflektion des eigenen Handelns und insbesondere dazu verpflichten, dessen Nutzen und Risiken gegeneinander abzuwägen und dabei das Gebot der Menschlichkeit ernst zu nehmen.^4^

## Footnotes

1 siehe dazu die verdienstvolle Quellenerschließung von [4].

2 Dieser Abschnitt basiert auf [1, 6, 7].

3 [17] Dies ist nur ein Beispiel der allgemeinen nationalsozialistischen Verformung der Normativität, wie sie u. a. von Herlinde Pauer-Studer und David Velleman ebenso eingehend wie eindrucksvoll beschrieben wurden: [18, 19].

4 Näheres zum Thema: Helmchen H (im Druck) Individuum und Zeitgeist. Wandel von Überzeugungen: Eugenik, Antipsychiatrie, Menschenrechte. V&Runipress.
